# Identification of *Leishmania donovani* PEX5-PTS1 Interaction Inhibitors through Fluorescence Polarization-Based High-Throughput Screening

**DOI:** 10.3390/molecules29081835

**Published:** 2024-04-17

**Authors:** Trong-Nhat Phan, Kyu-Ho Paul Park, David Shum, Joo Hwan No

**Affiliations:** 1 Institute of Applied Science and Technology, School of Technology, Van Lang University, Ho Chi Minh City 700000, Vietnam; nhat.pt@vlu.edu.vn; 2 Faculty of Applied Technology, School of Technology, Van Lang University, Ho Chi Minh City 700000, Vietnam; 3 Host-Parasite Research Laboratory, Discovery Biology, Institut Pasteur Korea, Seongnam-si 13488, Gyeonggi-do, Republic of Korea; 4 Screening Discovery Platform, Institut Pasteur Korea, Seongnam-si 13488, Gyeonggi-do, Republic of Korea; kyuhopaul.park@ip-korea.org (K.-H.P.P.); david.shum@ip-korea.org (D.S.)

**Keywords:** PEX5, peroxisomal targeting signal, *Leishmania*, inhibitor, high-throughput screening

## Abstract

Leishmaniasis, an infectious disease caused by pathogenic *Leishmania* parasites, affects millions of people in developing countries, and its re-emergence in developed countries, particularly in Europe, poses a growing public health concern. The limitations of current treatments and the absence of effective vaccines necessitate the development of novel therapeutics. In this study, we focused on identifying small molecule inhibitors which prevents the interaction between peroxin 5 (PEX5) and peroxisomal targeting signal 1 (PTS1), pivotal for kinetoplastid parasite survival. The *Leishmania donovani* PEX5, containing a C-terminal tetratricopeptide repeat (TPR) domain, was expressed and purified, followed by the quantification of kinetic parameters of PEX5-PTS1 interactions. A fluorescence polarization-based high-throughput screening assay was developed and small molecules inhibiting the *Ld*PEX5-PTS1 interaction were discovered through the screening of a library of 51,406 compounds. Based on the confirmatory assay, nine compounds showed half maximal inhibitory concentration (IC_50_) values ranging from 3.89 to 24.50 µM. In silico docking using a homology model of *Ld*PEX5 elucidated that the molecular interactions between *Ld*PEX5 and the inhibitors share amino acids critical for PTS1 binding. Notably, compound P20 showed potent activity against the growth of *L. donovani* promastigotes, *L. major* promastigotes, and *Trypanosoma brucei* blood stream form, with IC_50_ values of 12.16, 19.21, and 3.06 μM, respectively. The findings underscore the potential of targeting *Ld*PEX5-PTS1 interactions with small molecule inhibitors as a promising strategy for the discovery of new anti-parasitic compounds.

## 1. Introduction

Leishmaniasis is a neglected tropical disease caused by protozoan parasites of the genus *Leishmania*. Based on the clinical manifestations, the disease is classified into cutaneous, muco-cutaneous, and visceral leishmaniasis [1]. The estimated incidence for cutaneous leishmaniasis (CL) ranges from 0.7 to 1.2 million cases annually with most cases occurring in Central Asia, the Middle East, the Mediterranean basin in Europe and the Americas [2]. For visceral leishmaniasis (VL), around 100,000 cases are estimated per year mainly distributed in Brazil, India, Sudan, Ethiopia, and Kenya posing significant health challenges [2,3,4]. The disease is transmitted through the bite of the female sand fly in the genera of *Phlebotomus* or *Lutzomyia* for the New World and the Old World, respectively [5]. The clinical forms depend on the species of *Leishmania* transmitted by the sand fly in which *L. donovani* and *L. infantum* are responsible for VL, and *L. major* and *L. tropica* are the main causes for CL [6,7]. Once infected, patients are treated with therapeutic agents such as pentavalent antimony and amphotericin B. However, the use of these treatments is hindered by high costs, toxicity, adverse side effects, resistance issues, and limitations in the routes of administration [8,9,10,11,12]. Due to these reasons, there is a need for the identification and development of novel drugs and drug targets that are specific to leishmaniasis with enhanced pharmacological efficacy.

Glycosomes are unique organelles in *Leishmania* as well as in *Trypanosoma*, a protozoan parasite belonging to the same class of kinetoplastida causing Chagas disease or Human African Trypanosomiasis. They are responsible for glucose metabolism, which is essential for their survival, and are devoid of genomic material, relying on the import of matrix enzymes from the parasite’s cytosol for their functions [13,14,15,16,17]. The biogenesis of glycosomes primarily involves PEX proteins. Cytosolic enzymes are directed to their specific destination of glycosomes post-translationally, facilitated by either of the two peroxisomal targeting signals, PTS1 and PTS2, located at the C-terminus and N-terminus of proteins, respectively. And the C-terminal tetratricopeptide repeat (TPR) domain of PEX5 recognizes PTS1, typically characterized by a C-terminal tripeptide sequence SKL or its variants, while the PEX7 protein, containing a WD40 repeat, recognizes PTS2 cargo proteins [18,19,20]. The interaction of the cargo-loaded receptor PTS1-PEX5 with the membrane-associated PEX14 protein facilitates the formation of a dynamic, transient import pore, enabling the translocation of PTS1-cargo enzymes into the glycosomal matrix [21,22]. The depletion of PEX5 or PEX7 via RNA interference (RNAi) has demonstrated a profound impact on glycolysis and ATP production, crucial for the viability of *T. brucei* [23]. Also, RNAi-mediated knockdown of the peroxin PEX14 leads to the death of procyclic stage of *T. brucei* [24]. Moreover, the disruption of PEX14 and the N-terminal domain of PEX5 interaction by small molecule inhibitors has been shown to effectively kill *Trypanosoma* parasites both in vitro and in vivo by preventing the translocation of glycosomal proteins into glycosomes [25,26,27]. On the C-terminal side of PEX5, the site where the TPR domain is recognized by PTS1 in glycosomal proteins, is another potential site for inhibitor discovery. Recently, screening of approximately 30,000 compounds has yielded six hits that inhibit the *T. cruzi* PEX5-PTS1 interaction and have also demonstrated activity against *T. brucei* growth in vitro [28]. 

Most of the efforts around the discovery of pharmacological inhibitors against PEX14-PEX5-PTS1 axis have been limited to *Trypanosoma* parasites even though the machinery is present and found critical for the survival of *Leishmania*. Thus, in this study, we aimed to identify inhibitors of *Leishmania* C-terminal PEX5-PTS1 interaction using fluorescence polarization (FP)-based high-throughput screening (HTS). Some of identified compounds impeded the growth of *Leishmania* and *Trypanosoma* in vitro. Structural modeling was performed to predict the binding of these inhibitors to the *Ld*PEX5CT protein thereby gaining a molecular understanding of the mode of inhibition.

## 2. Results

### 2.1. Characterization of LdPEX5 and PTS1 Interaction and Inhibition with Peptides

**Figure 1:**
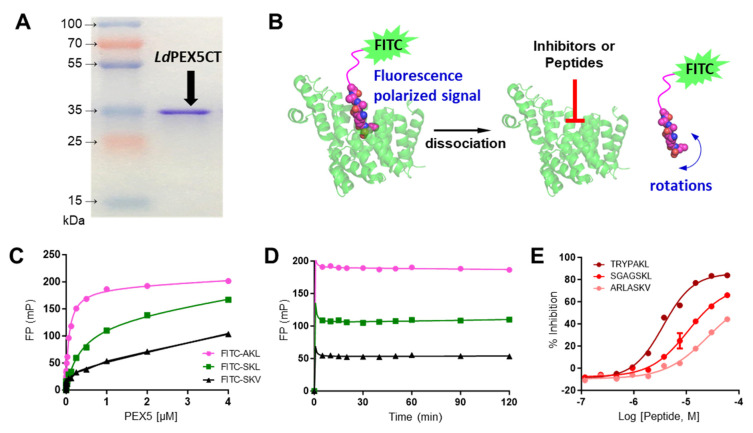
Purified *Ld*PEX5CT and characterization of different PTS1 peptides binding to *Ld*PEX5CT using fluorescence polarization method. (**A**) SDS-PAGE analysis of *Ld*PEX5CT protein fraction on an SDS-PAGE gel. (**B**) The schematics of fluorescence polarization assay developed with *Ld*PEXCT. (**C**) Three types of PTS1 peptides (5 nM, FITC-labeled) were titrated with 4 μM *Ld*PEX5CT (half dilution, 10 points), and the changes in FP were monitored. The binding constant (K_d_) of *Ld*PEX5 was calculated using GraphPad Prism with a binding–saturation equation. FITC-TRYPAKL (●), FITC-SGAGSKL (■), and FITC-ARLASKV (▲). (**D**) The stability of the FP signal was observed over 120 min for three labeled PTS1 peptides (5 nM) interacting with *Ld*PEX5 (1 μM). FITC-TRYPAKL (●), FITC-SGAGSKL (■), and FITC-ARLASKV (▲). (**E**) IC_50_ values for three unlabeled peptides as inhibitors were determined at 1 μM *Ld*PEX5 and 5 nM FITC-SGAGSKL, with 60 μM (half dilution, 10 points) of TRYPAKL (●), SGAGSKL (●), and ARLASKV (●).

The expression and purification of the *Ld*PEX5 protein resulted in a product with an apparent molecular mass of 35 kDa, as confirmed by SDS-PAGE (Figure 1A). The binding of FITC labeled PTS1 peptides to *Ld*PEX5 was measured through fluorescence polarization method (Figure 1B). Dissociation constant (K_d_) values for *Ld*PEX5 interacting with three labeled peptides—xanthine phosphoribosyltransferase (FITC-TRYPAKL), galactokinase (FITC-SGAGSKL), and phosphofructokinase (FITC-ARLASKV)—were determined. The K_d_ values for *Ld*PEX5 interaction were found to be 78 nM, 382 nM, and 102 nM for FITC-TRYPAKL, FITC-SGAGSKL, and FITC-ARLASKV, respectively (Figure 1C).

For HTS adaptation, the stability of the binding assay over time was examined. Using 1 µM *Ld*PEX5 and 5 nM of labeled probes, various incubation times up to 120 min were tested. As the binding rapidly reached equilibrium after 5 min, the incubation time of 60 min was selected as optimal for peptide testing and compound screening (Figure 1D). In the absence of a reference inhibitor for positive control, unlabeled PTS1 probes were tested as a control. Tests were conducted with a constant concentration of 1 µM *Ld*PEX5 and 5 nM labeled probe (FITC-SGAGSKL), and varying concentrations of unlabeled PTS1 probes (TRYPAKL, SGAGSKL, and ARLASKV) to determine their IC_50_ values. The IC_50_ values were 3.61 µM for unlabeled TRYPAKL, 10.65 µM for SGAGSKL, and >60 µM for ARLASKV (Figure 1E). Based on the K_d_ and IC_50_ values of labeled and unlabeled PTS1 peptides, labeled FITC-SGAGSKL was selected as the probe and unlabeled TRYPAKL as the positive control for HTS.

### 2.2. Identification of Small Molecule Inhibitor through FP-Based HTS

**Figure 2:**
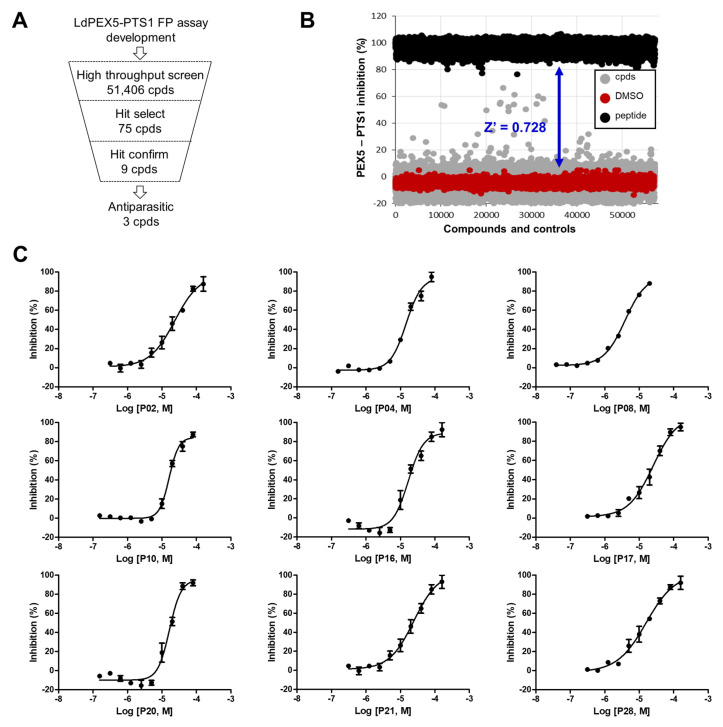
Identification of *Ld*PEX5-PTS1 interaction small molecule inhibitors through FP-based HTS and hit validation. (**A**) Flowchart depicting the process of identifying *Ld*PEX5-PTS1 interaction inhibitors via HTS. (**B**) Results of the FP-based HTS from an in-house library of 51,406 compounds. Red indicates the negative control (DMSO); black represents the positive control (TRYPAKL peptide); gray signifies tested compounds. (**C**) Dose–response curves of nine hits against *Ld*PEX5-PTS1 interaction. The results are expressed as mean standard deviation values for three independent experiments.

**Figure 3:**
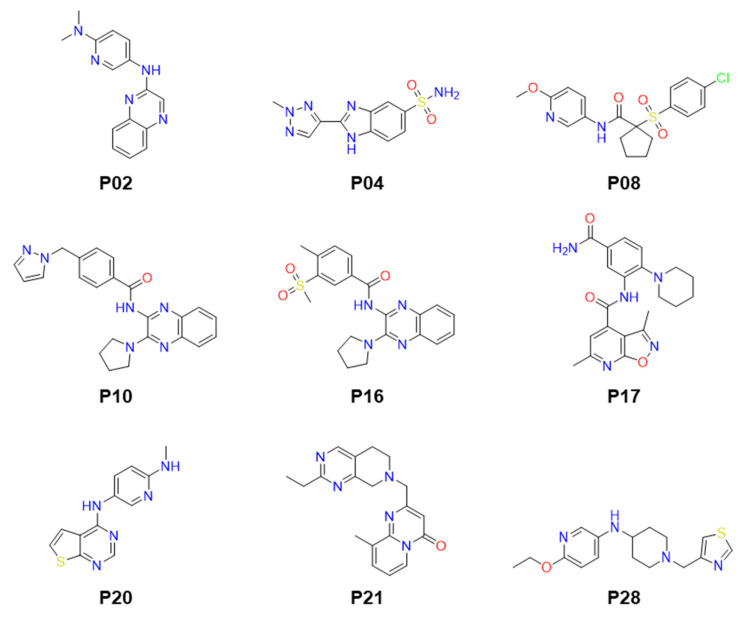
The structures of identified confirmed inhibitors.

The screening was conducted in a 384-well plate format, utilizing an in-house library of 51,406 compounds with diverse chemical structures (Figure 2A). These compounds were screened at a concentration of 20 µM. A Z’ value of 0.728 was achieved, indicating a robust HTS process with an excellent assay window (Figure 2B). Applying a threshold of greater than 20% inhibition, 75 compounds were identified as primary hits, resulting in a hit rate of 0.146% and further evaluated to determine IC_50_ values (Figure 2B). Out of these, nine compounds demonstrated the inhibition of the *Ld*PEX5-PTS1 interaction with IC_50_ values ranging from 3.89 ± 0.15 to 24.50 ± 1.05 µM (Figure 2C and Table 1). The structures of these compounds are presented in Figure 3. The structures of identified compounds were mostly distinct from each other, but P10 and P16 shared the N-(3-pyrrolidin-1-ylquinoxalin-2-yl)benzamide scaffold with similar IC_50_ values of ~16 µM.

### 2.3. Molecular Modeling of Inhibitor and LdPEX5 Interaction

**Figure 4:**
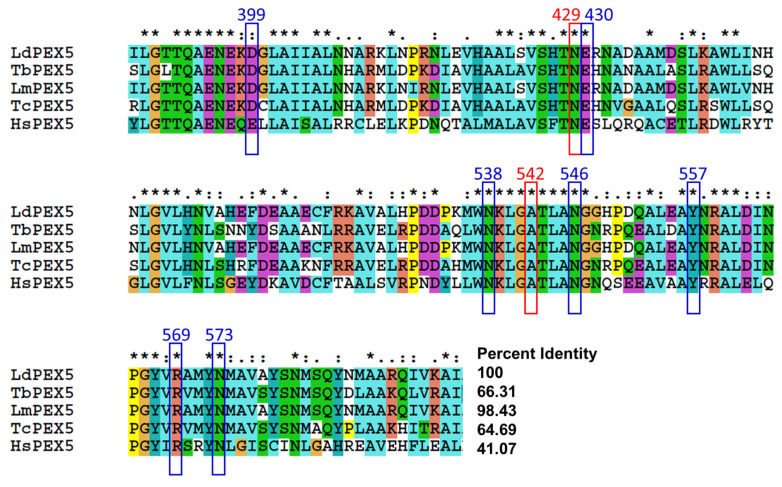
Multiple sequence alignment analysis of *Ld*PEX5 and PEX5 from other organisms. Nine amino acid residues involved in the interaction between PEX5 and PTS1 (SKL) are indicated as blue (hydrogen binding) and red (hydrophobic interaction) squares.

Multiple sequence alignment analysis was performed for *Ld*PEX5 in comparison with PEX5 proteins from other kinetoplastid parasites and human PEX5. *Ld*PEX5 showed high sequence identity with *L. major* PEX5 (98.43%), *T. brucei* PEX5 (66.31%), and *T. cruzi* PEX5 (64.69%) as expected, and a lower similarity with human PEX5 (41.07%) (Figure 4). According to Sampathkumar et al. [29], nine amino acids critical for the binding between *Tb*PEX5 and PTS1 (SKL) are conserved across kinetoplastid PEX5 and human PEX5. These nine residues involved in PEX5-PTS1 binding (D399, N429, E430, N538, A542, N546, N557, R569, and N573) were conserved in kinetoplastid PEX5 and a minor difference in D399 to E was observed in human PEX5 (Figure 4).

**Figure 5:**
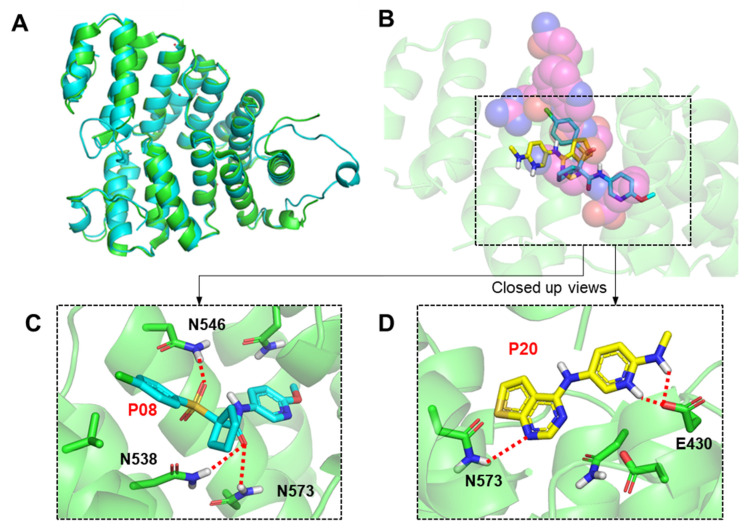
Modeling of *Ld*PEX5 domains and molecular docking predictions to predict the binding mode of inhibitors. (**A**) Structural alignment of *Ld*PEX5 and *Tb*PEX5 using PyMOL. The green cartoon represents the structure of *Ld*PEX5, and the cyan cartoon represents the structure of *Tb*PEX5. (**B**) Docking results show inhibitors (cyan: P08, yellow: P20) superimposed with SKL (in magenta) on the *Tb*PEX5 X-ray structure (PDB code: 3CVP). SKL binding sites are marked with black rectangles. (**C**) Close-up view of docking analysis illustrating potential interactions between *Ld*PEX5 and compound P08 (in cyan), and (**D**) compound P20 (in yellow). Dotted red lines indicate hydrogen bonds.

A homology model of *Ld*PEX5 was constructed using *Tb*PEX5 (PDB code: 3CVQ) as a template with a root mean square deviation (RMSD) of 0.509 Å (Figure 5A). The Ramachandran plot of the model indicates that 95.51% of amino acids are located within the favored region (Supplementary Figure S1). An analysis of the active sites identified seven residues involved in forming hydrogen bonds and two residues in hydrophobic interactions between *Tb*PEX5 and PTS1. A docking analysis indicated that compounds occupied the peptide (SKL motif) binding site of *Ld*PEX5 (Figure 5B). For compound P08 (1-((4-chlorophenyl)sulfonyl)-*N*-(6-methoxypyridin-3-yl)cyclopentane-1-carboxamide), which exhibited the highest inhibition against the *Ld*PEX5-PTS1 interaction, extended into the leucine site of SKL motif binding pocket through the methoxypyridine moiety. The oxygen in the carbonyl group interacted with the –NH of Asn538 and Asn573 through hydrogen bonding (3.0 and 2.9 Å; Figure 5C). Another oxygen in the sulfur dioxide group also engaged in hydrogen bonding with the –NH of Asn546 (2.7 Å; Figure 5C). The calculated docking score (GScore) of the compound was −6.35 kcal/mol. For P20 (N^2^-methyl-N^5^-(thieno [2,3-d]pyrimidin-4-yl)pyridine-2,5-diamine), the compounds with highest anti-parasitic activity described in the later section, occupies the lysine site of SKL motif binding pocket and the GScore from the docking was −5.68 kcal/mol. The oxygen in the carboxyl group of Glu430 formed hydrogen bonds with two –NH groups of the compound (1.6 and 2.7 Å; Figure 5D). Additionally, the –NH group of the thiophene pyrimidine in compound P20 formed a hydrogen bond with the –NH group of Asn573 (3.0 Å; Figure 5D). For P04 and P10, the predicted binding modes also align with the SKL motif of *Ld*PEX5 with the GScore of −5.00 and −4.52 kcal/mol, respectively (Supplementary Figure S2A). In the case of P04 (2-(2-methyl-2*H*-1,2,3-triazol-4-yl)-1*H*-benzo[*d*]imidazole-5-sulfonamide), notable interactions include hydrogen bonds between the –NH of N546 and the lone-pair electrons from the nitrogen atoms of the triazole (2.4 Å) and imidazole (2.4 Å) (Supplementary Figure S2B). For P10 (4-((1*H*-pyrazol-1-yl)methyl)-*N*-(3-(pyrrolidin-1-yl)quinoxalin-2-yl)), N546 is also found to be important for forming a hydrogen bond (2.3 Å) with the oxygen within the amide group of the compound, and K539 and Y572 were predicted to form π–cation (6.5 Å) and π–π (4.3 Å) interactions, respectively.

### 2.4. Anti-Parasitic Activity of Identified Inhibitors

**Figure 6:**
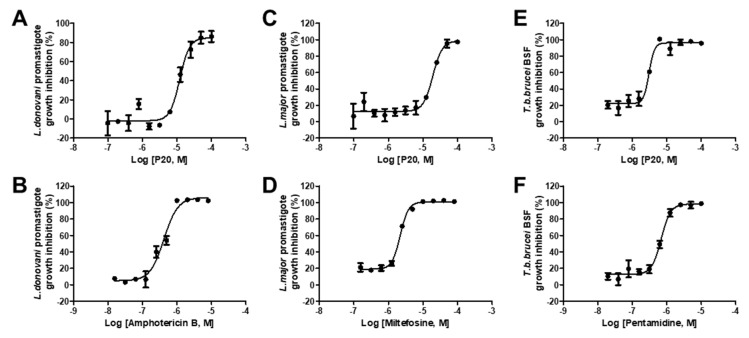
In vitro activity of identified inhibitors against kinetoplastid parasite growth. (**A**) Inhibition of *L. donovani* promastigote growth with P20 and the reference compound amphotericin B (**B**). (**C**) Inhibition of *L. major* promastigote growth with P20 and miltefosine (**D**). (**E**) Inhibition of *T. brucei* BSF growth with P20 and the reference compound pentamidine (**F**). The results are expressed as mean standard deviation values for three independent experiments.

The activities of nine hit compounds were assessed against *L. major* promastigotes, *L. donovani* promastigotes, and *T. brucei* BSF growth (Table 1). Among these, compound P20 exhibited inhibitory activity against all the tested parasites. The IC_50_ values were determined as 12.16 ± 1.36 µM for *L. major*, 19.21 ± 2.65 µM for *L. donovani*, and 3.06 ± 0.38 µM for *T. brucei* BSF (Figure 6). Compound P10 showed activity of IC_50_ = 3.3 ± 0.23 µM in *L. donovani* but was not active in other two parasites, and the structurally similar compound P16 with a similar inhibitory effect in PEX5-PTS1 interaction was not able to inhibit the growth of parasites. P20 and P10 were then tested against the intracellular amastigote form of Leishmania but were found to be inactive.

## 3. Discussion

Protein transport within peroxisomes is pivotal for Kinetoplastida parasites and has been identified as a valid therapeutic target for trypanosomiases. The PEX system, crucial for protein transport, comprises over a dozen distinct components, offering numerous potential targets for exploration in *Trypanosoma*. Recently, the PEX14-PEX5 protein–protein interaction, essential for glycosomal protein import, has been highlighted as an attractive target for *Trypanosoma* inhibitor discovery [25,26]. Furthermore, hit compounds that inhibit the interaction between *Tc*PEX5 and PTS1 cargo have been shown to effectively kill *T. brucei* parasites in cell culture [28]. Notably, despite belonging to the same kinetoplastid parasites group as *Trypanosoma*, no research has yet explored this interaction in *Leishmania* parasites.

In this study, fluorescence polarization-based high-throughput screening (FP-HTS) was employed to identify inhibitors of the *Ld*PEX5-PTS1 interaction. Prior to this study, investigations focusing on *Ld*PEX5-PTS1 interaction had not been reported, with only one study on *Tc*PEX5-PTS1 interaction, which identified 48 hits from HTS of a ~30,000 compound library resulting in a 0.16% hit rate [28]. The hit rate from this study was even lower (0.054%) possibly due to the differences in substrate concentrations, K_d_ values, or the structural features of compounds in the library. However, in general, the hit rate was relatively low in both PEX5 HTS studies. In terms of inhibitory capacity, the previously discovered inhibitors from *Tc*PEX5-PTS1 screening showed IC_50_ values ranging from 33 to 705 µM, whereas, in this study, the nine discovered compounds demonstrated IC_50_ values from 4 to 25 µM. Further molecular modeling studies characterized amino acids that are involved in the binding of inhibitors. The most potent compound P08 was shown to align on top of SKL motif binding position, where the bent conformation constrained by cyclopentane may have led to favorable positioning of the compounds. The sulfone and the amide group in P08 created interactions with N546, N538, and N573 of PEX5, which were also reported critical for the binding of SKL motif [29]. The 2-methoxypyridine moiety of P08 extended deep into the leucine site of SKL and, with the extra space available, the site may offer potential for compound optimizations. On the other hand, compound P20 was modeled to have a different binding mode to P08 and the compound extended more into the lysine site of SKL. Especially, the nitrogen in the 2-(methylamino)pyridine moiety of P20 was predicted to form a hydrogen bond with E430 of PEX5, an amino acid also responsible for interacting with the lysine of SKL. The structures of identified inhibitors from this study were largely different from the chemotypes discovered by Napolitan et al., where quinoxaline moieties and amide linkers were frequently found within the structures of identified inhibitors from this screen [28]. Nevertheless, in terms of interactions, the amino acids N546 and N573 from PEX5 were considered to form favorable interactions with discovered inhibitors from both studies [28].

The anti-parasitic assay demonstrated that compound P20 effectively inhibited the growth of *L. donovani*, *L. major*, and *T. brucei*. The IC_50_ value for *T. brucei* was 4–6 times higher compared to those for *L. donovani* and *L. major*. This result is particularly interesting since P20 was selected based on its ability to inhibit the PEX5-PTS1 interaction of *L. donovani*, yet it also exhibited anti-parasitic activity against *L. major* and, notably, *T. brucei*. This could be attributed to the high conservation of PEX5 proteins and PTS1 of cargo proteins among these kinetoplastid protozoans and, in particular, the amino acids responsible for P20 interaction with *Ld*PEX5 (E430 and N573) are conserved in *Lm*PEX5 and *Tb*PEX5. The previously identified six *Tc*PEX5-PTS1 interaction inhibitors showed IC_50_ values ranging from 2.18 to 12.4 µM against *T. brucei* and the P20 from this study had an IC_50_ of 3.06 µM showing comparable activity [28]. Even though the compound was active against three different kinetoplastid parasites, no activity was observed in the intracellular form of *Leishmania*. It is possible that penetrating an extra membrane of the host cell or the differing protonation states of the compound in the acidic environment of the parasitophorous vacuole, where the amastigotes reside, could be potential reasons for the inactivity of the compounds against the intracellular form of *Leishmania*. Compound P10 was found to show the most potent activity against *L. donovani* promastigotes with an IC_50_ values of 3.3 µM; however, most other confirmed hit compounds identified from the PEX5-PTS1 screening were inactive against parasite growth, possibly due to various factors such as membrane permeability. For instance, a report on predicting the antimicrobial activity of compounds in relation to their target protein inhibition and chemical descriptors have shown that the solubility terms (logP, SloP, logS), which affect membrane permeability, have been identified as major components involved in translating activities from the target to the microbes [30].

In summary, a large-scale screening campaign targeting the *Ld*PEX5-PTS1 interaction was conducted using an FP-based HTS system. Several compounds showed the inhibition of the interaction with activity against kinetoplastid parasites. Docking studies suggest critical residues and interactions between PEX5 and the inhibitors. Since the discovered inhibitors share similar chemical moieties and interactions, further optimization of inhibitors through structure–activity relationship studies will guide toward generating inhibitors with improved potency against the PEX5-PTS1 interaction as well as the parasites in vitro.

## 4. Materials and Methods

### 4.1. Reagents and Parasites

All the restriction enzymes used in this study were sourced from New England BioLabs (240 County Road Ipswich, Ipswich, MA, USA). The PTS1 peptides, both unlabeled and fluorescently labeled at the N-terminus with fluorescein isothiocyanate (FITC), were purchased from Peptron (Daejeon, Republic of Korea). All the inhibitors were purchased from Enamine (Kyiv, Ukraine). *L. donovani* strain MHOM/SD/62/1S-CL2D and *L. major* strain LV39 (MRHO/Sv/59/P) were cultured in their extracellular promastigote forms. The parasite was cultured at 28 °C using M199 medium (Sigma-Aldrich, St. Louis, MO, USA) supplemented with 40 mM HEPES, 0.1 mM adenine, 0.0001% biotin, and 4.62 mM NaHCO_3_, along with additional supplements of hemin and xanthine. The *T. brucei brucei* Lister strain 427 in its bloodstream form (BSF) was cultured at 37 °C in a controlled atmosphere consisting of 5% CO_2_ in HMI-9 medium enriched with 10% fetal bovine serum (FBS), 100 µg/mL penicillin, and 100 µg/mL streptomycin. The parasites were maintained through only 10 passages for this study.

### 4.2. Expression and Purification of His_6_-LdPEX5CT

The C-terminal *Ld*PEX5 expression plasmid was constructed by cloning the *Ld*PEX5CT open reading frame into the pET15b vector, incorporating a 6×-histidine affinity tag at the amino terminus (Novagen, Madison, WI, USA). The fragment comprising a 966 bp DNA segment encoding *Ld*PEX5CT, corresponding to 322 amino acids (AF198051.1: 303-625), was synthesized (Genscript, Piscataway, NJ, USA) and cloned into the *NdeI*/*BamHI* sites of the pET15 vector using restriction enzymes (New England Biolabs, Ipswich, MA, USA). *Escherichia coli* BL21 (DE3) cells transformed with pET15b-His6-*Ld*PEX5CT were cultured in LB medium supplemented with 100 µg/mL ampicillin. Protein expression was induced with 0.1 mM isopropyl β-d-1-thiogalactopyranoside (IPTG) and the culture was maintained at 16 °C for 14 h. Cell pellets from 3 L of bacterial culture were collected and then lysed by sonication, and the proteins were isolated using a 16/10 HisPur^®^ Ni^2+^-NTA resin column (Thermo Scientific, Waltham, MA, USA) on an AKTA Explorer chromatography system, following the methodology published by Phan et al. [31]. The eluted fractions were analyzed via SDS-PAGE confirming over 95% purity. These fractions were then dialyzed against a buffer containing 50 mM Tris-Cl (pH 8.0), 150 mM NaCl, and 10% glycerol. The concentration of LdPEX5CT was quantified using the Pierce^TM^ Braford Protein Assay kit (Thermo Fisher Scientific, Waltham, MA, USA) with bovine serum albumin (BSA) as the standard. The purified protein was stored at −80 °C in a solution containing 40% glycerol, to be used for the HTS.

### 4.3. LdPEX5-PTS1 Peptides Interaction Assay

For the interaction experiments between *Ld*PEX5 and three labelled peptides (FITC-TRYPAKL, FITC-SGAGSKL, and FITC-ARLASKV), 5 nM of each peptide was combined with 4 µM of *Ld*PEX5 in a half dilution series (10 points) within a total volume of 50 µL of assay buffer. The assay buffer was 15 mM KH_2_PO_4_ (pH 7.2), 5% glycerol, 0.05% Tween-20, and 1 mg/mL BSA. The interaction between *Ld*PEX5 and the labeled peptides was monitored using changes in fluorescence polarization induced upon binding of *Ld*PEX5 to the fluorescein-labeled peptides. Fluorescence readings were performed using Envision equipment with an excitation wavelength of 485 nm and an emission wavelength of 535 nm. This assay was employed to calculate the binding constant (K_d_) of *Ld*PEX5 for its association with the labeled peptides. Data analysis was performed using GraphPad Prism software, utilizing a binding–saturation equation to represent a one-site total ligand binding model. The FP values were calculated using the formula FP = FP_min_ + [(FP_max_ − FP_min_) × C]/(K_d_ + C), where FP represents the measured fluorescence polarization, FP_min_ is the FP value of the PTS1 peptide alone, FP_max_ is the maximum FP value at saturation, K_d_ is the dissociation constant, and C is the concentration of the *Ld*PEX5 protein.

### 4.4. FP-Based HTS for LdPEX5-PTS1 Interaction Inhibitors

HTS using FP was conducted to identify inhibitors of the *Ld*PEX5-PTS1 interaction. The screening utilized an in-house compound library comprising 51,406 compounds with diverse structural profiles. All compounds were initially solubilized in 100% DMSO with a concentration of 2 mM, and the final testing concentration in the assay was set at 20 μM. DMSO served as the negative control, while unlabeled PTS1 peptide (TRYPAKL) was used as the positive control. The assay mixture included FITC-SGAGSKL peptide and *Ld*PEX5 at final concentrations of 5 nM and 1 μM, respectively. The reaction plates with compounds were incubated for 1 h at room temperature before FP signal detection. Single-point inhibition assays were performed, and the Z’ factor was employed to assess the quality of the assay. The Z’ factor was calculated using the formula Z′ = 1 − 3(σ_p_ + σ_n_)/(μ_p_ − μ_n_), where σ_p_ and σ_n_ are the standard deviations of the positive and negative controls, respectively, and μ_p_ and μ_n_ represent their respective mean values. The hits identified from the screening were confirmed by testing in a 10-point, 1/2 dilution manner, and the highest tested concentration varied depending on the solubility of the compounds.

### 4.5. Molecular Modelling Study

The protein sequences for *L. donovani* PEX5 (AAF67841.1), *L. major* PEX5 (XP_003722535.1), *T. brucei* PEX5 (AAD54220.1), *T. cruzi* PEX5 (PBJ69826.1), and *Homo sapiens* PEX5 (ALQ33751.1) were retrieved from the NCBI Protein Database (https://www.ncbi.nlm.nih.gov/, accessed on 10 March 2024). Sequence alignment was performed using ClustalX 2.1 and the alignment tool available on UniProt (https://www.uniprot.org/align/, accessed on 10 March 2024). The homology model of *Ld*PEX5 was constructed using SWISS-MODEL (http://swissmodel.expasy.org, accessed on 10 March 2024), with the crystal structure of *Tb*PEX5 (PDB code: 3CVP) as the template. Docking analyses were conducted using the Glide docking module in the Maestro software suite version 13.9 (Schrödinger). Hydrogen atoms were added using the Protein Preparation Wizard, followed by energy minimizations (Hydrogen only), and the docking grid was generated encompassing the SKL motif binding site. The docking was performed with XP mode using the standard parameters, retaining 10 poses per ligand. The poses were selected based on Glide Score (<−4 kcal/mol) and visual inspections. Structural alignments and visualization of the modeling results were carried out using PyMOL version 1.3 (The PyMOL Molecular Graphics System).

### 4.6. Anti-Parasitic Activity Assessment

The inhibitory activity of the identified inhibitors was assessed against *L. donovani* promastigotes, *L. major* promastigotes, and *T. brucei* BSF growth, following the methodology published by Phan et al. [32]. Briefly, the assays were conducted in 384-well plates, each well was seeded with 5 × 10^4^ parasites, and the compounds were exposed for 72 h. The compounds were tested in dose-diluted format, starting from 100 μM with a two-fold dilution across 10 points. Subsequently, resazurin was added to each well to achieve a final concentration of 200 μM. The plates were then incubated for an additional 5 h before fixation. The assay results were recorded using a Victor3 plate reader (PerkinElmer, Shelton, CT, USA). For the assessment against *L. donovani* intracellular amastigotes, phorbol 12-myristate 13-acetate-treated THP-1 cells were seeded at 0.8 × 10^4^ cells per well in a 384-well plate in RPMI-1640 complete medium supplemented with 10% FBS. After 48 h, the infection was carried out using *L. donovani* promastigotes with a multiplicity of infection of 10:1. The infected cells were treated with compounds for 72 h, then the cells and parasites were washed, stained with 5 µM 4′,6-diamidino-2-phenylindole (DAPI), and fixed with 4% paraformaldehyde. Images were acquired using Operetta^®^ CLS™ (PerkinElmer, Shelton, CT, USA), and the numbers of parasites and host cells were quantified using Columbus™ software version 2.8 (PerkinElmer, Shelton, CT, USA).

### 4.7. Statistical Analysis

All half-maximal inhibitory concentration (IC_50_) values were determined based on data obtained from three independent experiments. The dose–response curves were generated and analyzed using GraphPad Prism 6 software (GraphPad Software, San Diego, CA, USA). For the analysis, a sigmoidal dose–response equation was employed, incorporating a variable hill slope option.

## Figures and Tables

**Table 1:** Identified small molecules with inhibition values in *Ld*PEX5-PTS1 interaction and parasite growth assays.

#	Compounds	*Ld*PEX5-PTS1 Inhibition IC_50_ ± SD ^a^ (µM)	*L. donovani* IC_50_ ± SD ^a^ (µM)	*L. major* IC_50_ ± SD ^a^ (µM)	*T. brucei* IC_50_ ± SD ^a^ (µM)
1	P02	23.64 ± 0.86	>50	>50	>50
2	P04	14.61 ± 0.76	>50	>50	12.05 ± 2.19
3	P08	3.89 ± 0.15	>50	>50	>50
4	P10	16.18 ± 0.92	3.3 ± 0.23	>50	>50
5	P16	16.93 ± 1.03	>50	>50	>50
6	P17	25.23 ± 0.89	>50	>50	>50
7	P20	16.53 ± 0.79	12.16 ± 1.36	19.21 ± 2.65	3.06 ± 0.38
8	P21	24.50 ± 1.05	>50	>50	>50
9	P28	15.98 ± 0.96	>50	>50	>50
10	Amphotericin B	NA	0.42 ± 0.03	NA	NA
11	Miltefosine	NA	NA	7.37 ± 1.67	NA
12	Pentamidine	NA	NA	NA	0.69 ± 0.05

^a^ Mean half-inhibition concentrations (IC_50_) ± SD (standard deviations) of data from three independent replicate measurements are shown; NA, not applicable.

## Data Availability

No privacy or ethical restriction is required for this research data.

## Supplementary Materials

The following supporting information can be downloaded at: https://www.mdpi.com/article/10.3390/molecules29081835/s1, Figure S1: The Ramachandran plot of the LdPEX5CT model generated from Swiss-model. The plot indicates that 95.51% of amino acids are located inside the Ramachandran favored region.; Figure S2: The predicted binding mode of compounds P04 and P10 to the LdPEX5 homology model. (A) Overlay of the docked poses of P04 and P10 on top of the SKL motif from the TbPEX5 x-ray structure (PDB: 3CVP). The docking pose of compound P04 (B) and P10 (C) with molecular interactions indicated by red dotted lines.
