# Supplementary figures and images for: Identification of Leishmania donovani PEX5-PTS1 Interaction Inhibitors through Fluorescence Polarization-Based High-Throughput Screening
Source: Molecules. 2024 Apr 17;29(8):1835. doi: 10.3390/molecules29081835 (PMC11054337; doi:10.3390/molecules29081835)

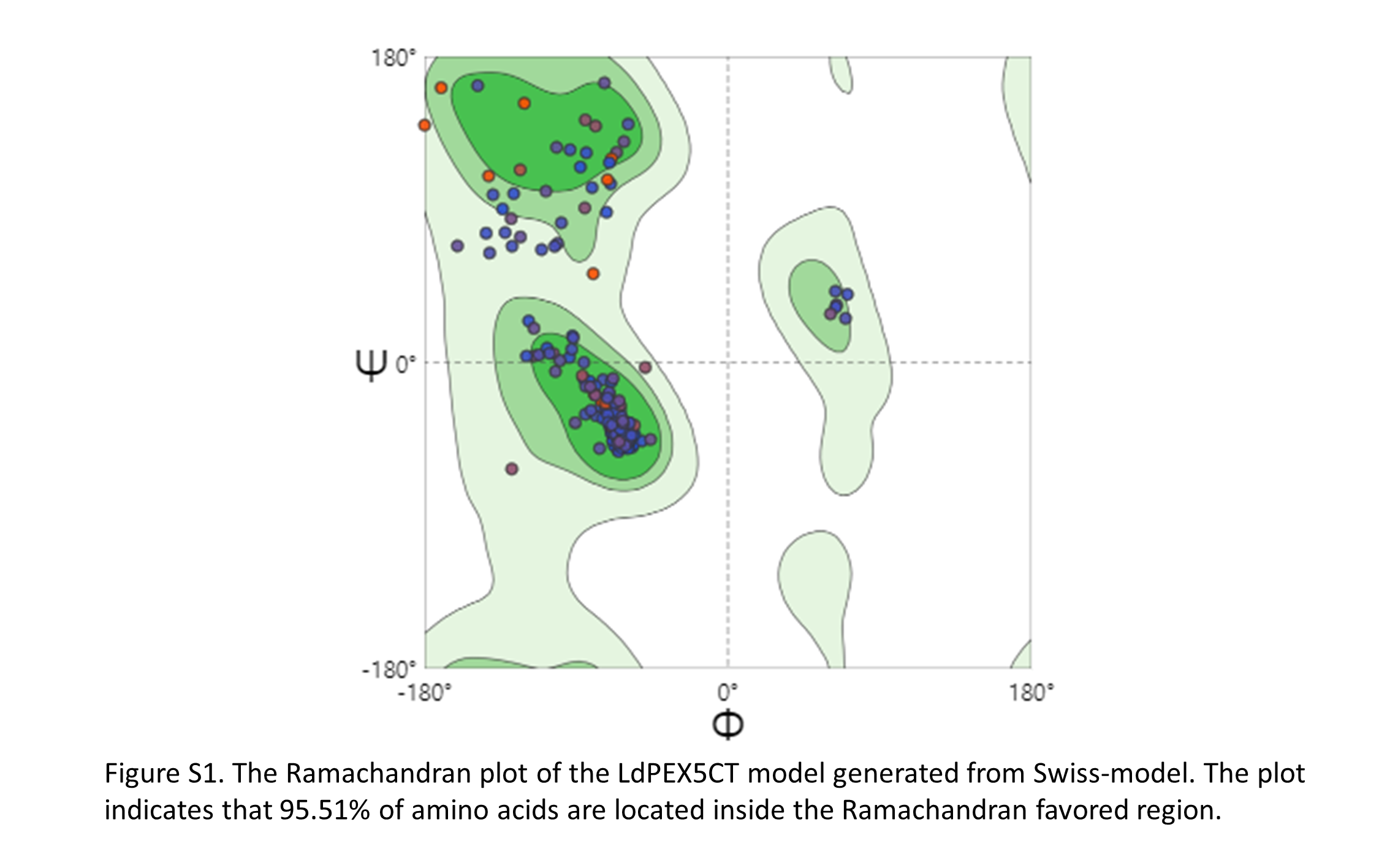

Supplement: Supplementary file 1 [file molecules-29-01835-s001.zip › LdPEX5_SI Figure S1.tif]

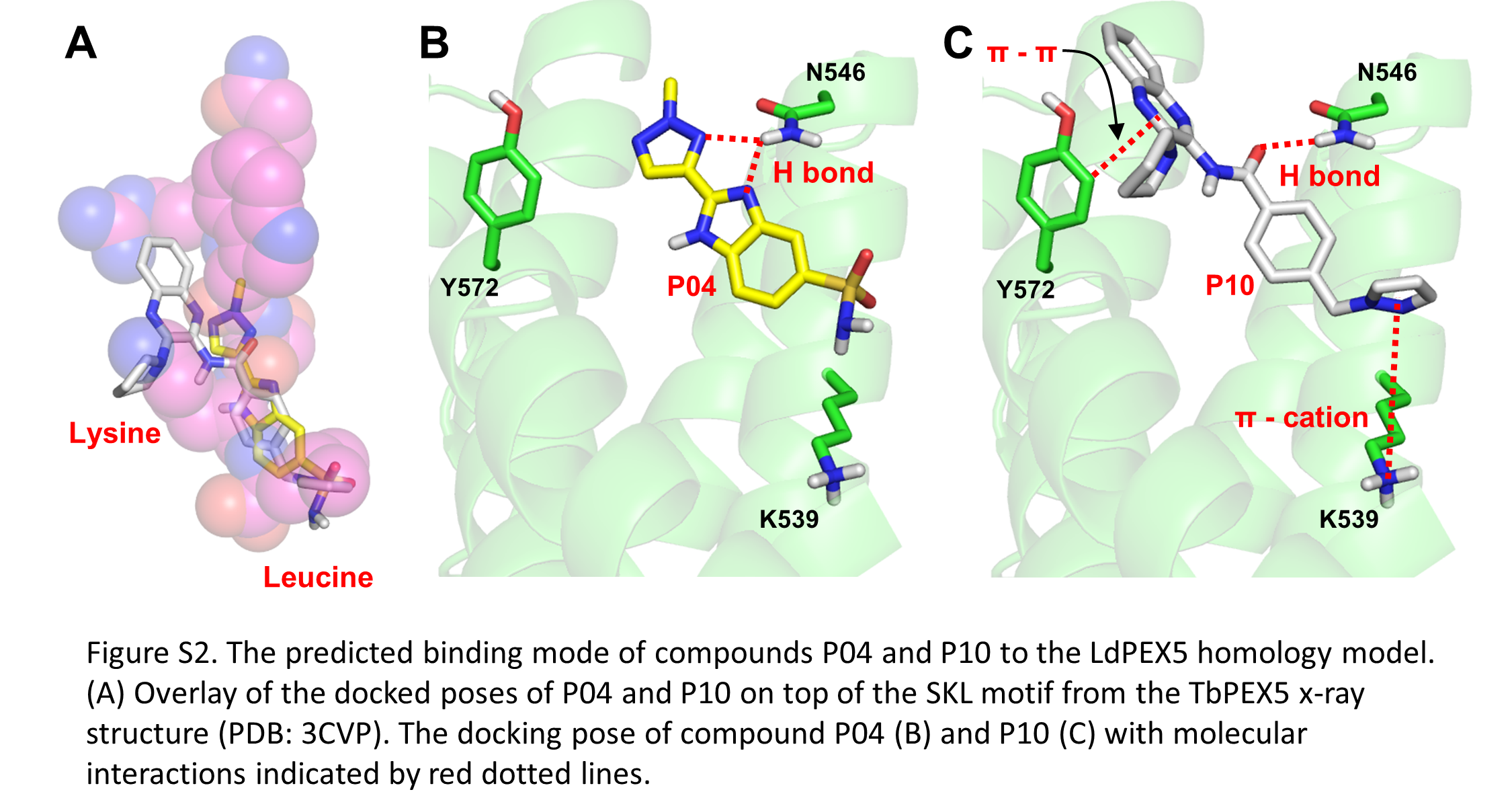

Supplement: Supplementary file 1 [file molecules-29-01835-s001.zip › LdPEX5_SI Figure S2.tif]
